# Drug therapy for the prevention and treatment of bronchopulmonary dysplasia

**DOI:** 10.3389/fphar.2015.00012

**Published:** 2015-02-16

**Authors:** Anjali Iyengar, Jonathan M. Davis

**Affiliations:** Department of Pediatrics, Floating Hospital for Children at Tufts Medical CenterBoston, MA, USA

**Keywords:** bronchopulmonary dysplasia, bronchodilators, corticosteroids, diuretics, lung injury

## Abstract

**Introduction:** As more infants are surviving at younger gestational ages, bronchopulmonary dysplasia (BPD) remains as a frequent neonatal complication occurring after preterm birth. The multifactorial nature of the disease process makes BPD a challenging condition to treat. While multiple pharmacologic therapies have been investigated over the past two decades, there have been limited advances in the field. Often multiple therapies are used concurrently without clear evidence of efficacy, with potential for significant side effects from drug-drug interactions.

**Methods:** Systematic literature review.

**Conclusion:** Although there is physiologic rationale for the use of many of these therapies, none of them has single-handedly altered the incidence, severity, or progression of BPD. Future research should focus on developing clinically significant end-points (short and long term respiratory assessments), investigating biomarkers that accurately predict risk and progression of disease, and creating appropriate stratification models of BPD severity. Applying a multi-modal approach to the study of new and existing drugs should be the most effective way of establishing the optimal prevention and treatment regimens for BPD.

## INTRODUCTION

Bronchopulmonary dysplasia (BPD) has been traditionally defined as a chronic form of lung disease in neonates treated with oxygen and positive pressure ventilation for a primary lung disorder. As more neonates at the threshold of viability are surviving, BPD continues to be a persistent and prevalent NICU morbidity with approximately 15,000 neonates diagnosed in the United States each year. BPD also carries a higher risk of neurodevelopmental morbidity and (Ehrenkranz et al., 2005) mortality, making it an extremely important complication of neonatal intensive care.

Bronchopulmonary dysplasia was first described by Northway et al. (1967) as chronic lung disease that developed in premature infants who were ventilated with high pressures and concentrations of oxygen. The histological appearance was characterized by parenchymal fibrosis, inflammation, and smooth muscle hypertrophy resulting in diffuse airway damage (O’Brodovich and Mellins, 1985). However, the nature of BPD has evolved into a “new” form of BPD typically seen in neonates surviving at the threshold of viability and characterized primarily by arrest of alveolar and vascular development (Husain et al., 1998; Jobe, 1999; Jobe and Bancalari, 2001; Baraldi and Filippone, 2007). The definition of BPD has also changed over time, with an NIH consensus conference capturing criteria from previous definitions and incorporating a stratification system based on clinical severity (Jobe and Bancalari, 2001; **Table 1**). Since the pathogenesis of BPD is multifactorial (e.g., mechanical ventilation, oxygen exposure, nutritional deficits, fetal growth restriction, and genetic susceptibility), a multidisciplinary approach is necessary for effective treatment. This article will discuss current pharmacologic approaches, which have not changed dramatically in the last 20 years. Often, many of these therapies are used concurrently with inadequate studies to define efficacy as well as the potential for significant side effects (especially drug-drug interactions). Finally, experimental agents that are being investigated for the prevention of BPD and associated complications of this common neonatal disease will be introduced.

**Table 1:** NIH severity-based diagnostic criteria for bronchopulmonary dysplasia (BPD).

Gestational age	<32 weeks
Timing of assessment	∙ 36 weeks post-menstrual age (PMA) or discharge home, whichever comes first ∙ Therapy with oxygen >21% for at least 28 days plus
Mild BPD	Breathing room air
Moderate BPD	Need for <30% oxygen*
Severe BPD	Need for ≥30% oxygen and/or positive pressure (PPV or CPAP)*

*A physiologic test confirming an oxygen requirement at the assessment time point remains to be defined. The test may include a pulse oximetry saturation range by Jobe and Bancalari (2001).

## METHODS

Approximately 100 articles, including animal studies, human pilot studies, randomized controlled trials (RCTs), meta-analyses, and systematic reviews published on the PubMed database were evaluated for inclusion in this article. Additionally, *ClinicalTrials.gov* was queried for ongoing studies investigating pharmacologic therapies for BPD.

## DIURETICS

Large volumes of intravenous fluids are often administered to premature neonates to provide adequate hydration and nutrition. Excessive fluid administration can be associated with pulmonary edema (especially with acute lung injury) and lead to increased respiratory support and ultimately BPD (Oh et al., 2005). Furosemide acts on the ascending loop of Henle and blocks chloride transport. Additionally, furosemide decreases interstitial edema and pulmonary vascular resistance and increases plasma oncotic pressure and lymphatic flow. It is the treatment of choice for fluid overload in BPD. Several studies have demonstrated alternate-day, daily, and even aerosolized furosemide improve clinical respiratory status, pulmonary mechanics, oxygenation, and facilitate weaning from mechanical ventilation. However, long-term benefits have not been established in infants with BPD (Rush et al., 1990; Sahni and Phelps, 2011; Stewart and Brion, 2011; Segar, 2012). Thiazides affect the renal tubular excretion of electrolytes but are less potent than loop diuretics. Potassium and bicarbonate excretion also occur which has prompted the use of thiazides in conjunction with spironolactone, a competitive inhibitor of aldosterone. This weak potassium-sparing diuretic facilitates sodium, chloride, and water excretion. A small number of controlled trials examining the use of thiazide diuretics and spironolactone in BPD have generated mixed results with urine output increasing, but not always accompanied by improvements in pulmonary mechanics (Engelhardt et al., 1989; Hoffman et al., 2000). The use of spironolactone does appear to offer any substantial benefit and is not recommended. Overall, diuretics offer short-term improvements in pulmonary mechanics but are associated with a number of side effects that may limit longer-term use (e.g., ototoxicity, electrolyte disturbances, azotemia, etc.). Furthermore, there are limited data demonstrating significant benefits of these agents when more meaningful outcome measures are analyzed such as reduction in the duration of mechanical ventilation and hospitalization or improved long-term clinical outcomes (less asthma, pulmonary infections, etc.). Additional longer-term studies are needed to establish optimal treatment regimens in infants with established BPD.

## BRONCHODILATORS

Albuterol (also known as ‘Salbutamol’) is an inhaled β_2_-agonist that is the recommended for the treatment of BPD with a strong component of reversible bronchospasm (Davis and Rosenfeld, 2005). It has been associated with short-term improvements in pulmonary resistance and lung compliance secondary to bronchial smooth muscle relaxation (Wilkie and Bryan, 1987). While a Cochrane review examining the role of albuterol was unable to find sufficient evidence of efficacy in the prevention of BPD, other studies have shown improvement in pulmonary mechanics following treatment (Robin et al., 2004; Ng et al., 2012). In summary, long-term efficacy has not been established and tolerance may develop with prolonged use.

Ipratropium bromide is a muscarinic antagonist that produces bronchodilation in chronically ventilated infants with BPD. Significant improvements in airway resistance and compliance has been shown in its isolated use or combined with a β_2_-agonist (Brundage et al., 1990). However, clinical trials have not demonstrated changes in the natural progression of BPD or long-term clinical respiratory status (De Boeck et al., 1998; Pantalitschka and Poets, 2006). Despite these findings, infants with BPD who develop wheezing may warrant a trial with albuterol initially with the addition of ipratropium bromide if significant side effects occur or clinical improvement isn’t seen with a β_2_-agonist alone.

## VITAMIN A

Vitamin A (i.e., retinol) is important in maintaining cell integrity and promoting tissue repair with deficiencies producing significant changes in the tracheobronchial tree (Anzano et al., 1980). Multiple studies have demonstrated that very low birth weight infants are deficient in Vitamin A and at a propensity to develop BPD (Shenai et al., 1990; Darlow and Graham, 2011). A landmark, multicenter Neonatal Research Network (NRN) trial investigated the benefits of vitamin A supplementation in improving survival without BPD in 807 neonates weighing <1000 g at birth. Intramuscular doses of 5000 IU of Vitamin A given three times a week for 4 weeks demonstrated a small (9%), but significant reduction in survival without chronic lung disease at 36 weeks post-menstrual age (PMA; Tyson et al., 1999). No increased toxicity was seen with the higher dosing regimen compared to placebo. However, long-term follow-up of these infants at 18–22 months could not demonstrate any improvement in mortality, neurodevelopmental impairment, or respiratory outcomes from treatment with Vitamin A (Ambalavanan et al., 2005). However, this study was not powered for demonstrating differences in these longer-term outcomes, so many centers still administer vitamin A routinely in infants at high risk for developing BPD.

## METHYLXANTHINES

Caffeine treatment for the prevention of apnea of prematurity and BPD is currently the standard of care in most neonatal intensive care units (Ghanta et al., 2013). It has been shown to increase respiratory drive, diaphragm contractility, and pulmonary compliance while reducing airway resistance (Davis et al., 1989; Aranda et al., 2010). These effects are of particular importance in chronically ventilated neonates who can develop skeletal muscle and diaphragmatic atrophy and fatigue. The improved muscle contractility may stabilize the chest wall and improve functional residual capacity facilitating successful extubation (Davis and Rosenfeld, 2005). Schmidt et al. (2006) conducted a large, multicenter RCT investigating the effects of caffeine on apnea of prematurity in a cohort of infants weighing 500–1250 g at birth. While infants in the treatment group had significantly less apnea of prematurity, they were also noted to have less BPD (defined as need for supplemental oxygen at 36 weeks PMA), patent ductus arteriosus (PDA), and cerebral palsy when followed out to 18–21 months corrected gestational age (Schmidt et al., 2007). However, these outcomes did not translate into longer-term benefits when this same cohort of infants was examined at 5 years of age (Schmidt et al., 2012). Despite these findings, caffeine therapy remains a standard medical approach to the prevention and treatmentof BPD.

Pentoxifylline is a methylxanthine derivative and phosphodiesterase inhibitor with immunomodulatory and anti-fibrotic properties (Almario et al., 2012; Ghanta et al., 2013). It has been proposed to have a therapeutic role in attenuating tissue injury associated with sepsis (Harris et al., 2000; Michetti et al., 2003). In a hyperoxia-induced lung injury model of BPD, pentoxifylline reduced lung edema and inflammatory cell infiltration while improving antioxidant activity, vascular development, and overall survival (Almario et al., 2012). A RCT in 150 very low birth weight infants demonstrated a reduction in BPD in infants receiving pentoxifylline compared to placebo (Ruszard et al., 2006). However, there is still insufficient evidence to support widespread usage and further safety and efficacy data is needed.

## CORTICOSTEROIDS

Marked inflammation in the lung appears to play an important role in the pathogenesis of BPD, unifying many factors into a single common pathway. Therefore, it is reasonable to consider the use of corticosteroids in treating BPD. The use of corticosteroids can be further delineated based on route of administration.

### SYSTEMIC

Historically, reviews of the use of systemic corticosteroids have investigated the effects of dexamethasone on BPD (treatment of existing lung injury as well as prevention) when administered during different time periods: early (<96 h after birth), moderately early (7–14 days after birth), or late (>3 weeks after birth; Halliday et al., 2003a,b,c). However, more recent reviews have classified trials as early (<7 days of life) or late (≥7 days after birth) based on timing of dexamethasone administration (Doyle et al., 2014a,b). All of these reviews have shown that dexamethasone facilitates extubation, reduces the combined endpoint of death or BPD at 28 days or 36 weeks PMA, and also reduces the incidence of PDA and ROP. However, the meta-analyses investigating trials where glucocorticoids have been used early in life have also found a significant increase in adverse long-term neurologic outcomes, specifically cerebral palsy (Doyle et al., 2014a). In contrast, there has been no significant increase in long-term neurologic outcomes detected with regard to moderately early and late administration of glucocorticoids (Halliday et al., 2003a,c; Doyle et al., 2014a). Nevertheless, the trend toward an increase in the incidence of abnormal neurologic findings in trials of late administration of glucocorticoids have prompted the American Academy of Pediatrics to issue a policy statement advising against the early use of dexamethasone and strongly recommending caution with the routine use of dexamethasone after 7 days of life (Watterberg, 2010). However, data from 16 RCTs extracted by Onland et al. (2009) demonstrated that moderately early administration of dexamethasone (7–14 days after birth) did not significantly increase the combined outcome of death or cerebral palsy and actually showed a dose dependent decrease (6.2%) in cerebral palsy with each incremental mg/kg increase in cumulative dexamethasone dose. Interestingly enough, this promising dose-dependent effect on neurodevelopmental outcome was not demonstrated in the delayed (>3 weeks) glucocorticoid treatment trials. These data illustrate the potential time-sensitive effects of dexamethasone and the need for clinicians to balance the known impact on neurodevelopmental outcome associated with prolonged mechanical ventilation and the development of BPD with the risks/benefits of systemic glucocorticoid treatment.

Other investigators have suggested that a primary cortisol deficiency in preterm infants increases the risk of BPD which may be amenable to early treatment with a less potent corticosteroid such as hydrocortisone (Watterberg, 2007). A meta-analysis from Doyle et al. (2010) evaluated eight RCT investigating the clinical effects of postnatal hydrocortisone given in the first week of life to VLBW infants. Infants who received hydrocortisone did not demonstrate a significant reduction in mortality, BPD, or cerebral palsy and actually had a significant increase in the incidence of gastrointestinal perforation (although this occurred more often when indomethacin was given concurrently). Indeed there has been emerging literature indicating that prolonged hydrocortisone exposure can negatively impact language and motor skills in the first years of life (Patra et al., 2014). Ongoing randomized, controlled clinical trials will no doubt help generate data on the appropriate dose and timing of hydrocortisone treatment for the prevention of BPD ^1^ (Onland et al., 2011).

### INHALED

Inhaled steroids have been examined as a therapeutic approach to the treatment of BPD in order to promote respiratory benefits while minimizing systemic side effects. Studies examining the benefits of inhaled corticosteroids administered early or late have not been able to demonstrate any impact of inhaled corticosteroids on short-term respiratory outcomes (e.g., death or BPD at 36 weeks PMA) or longer-term clinical respiratory status (Onland et al., 2012; Shah et al., 2012b). Additionally, inhaled corticosteroids appear to offer no clinical advantage over systemic steroid therapy (Shah et al., 2012a). The potential for systemic absorption of inhaled steroids and subsequent side effects (e.g., growth, adrenal suppression, etc.) warrants careful consideration before initiation of this treatment approach. Further research is needed to evaluate the type of inhaled steroid, timing, formulation, dosage, and method of administration that is most appropriate for the prevention and treatment of BPD.

## PULMONARY VASODILATORS

### INHALED

It is well-recognized that infants with BPD can experience intermittent episodes of hypoxia which can promote secondary pulmonary vasoconstriction and pulmonary hypertension, adding to the complexity of BPD (Khemani et al., 2007; Steinhorn, 2013). This has resulted in much interest in the selective pulmonary vasodilator nitric oxide (NO) as alterations in NO signaling, vascular growth, and reactivity appear to play a role in the development of BPD (MacRitchie et al., 2001; Afshar et al., 2003). In animal models of BPD, inhaled NO promotes pulmonary angiogenesis, reduces inflammation, and decreases apoptosis and oxidant damage (Gutierrez et al., 1996; Balasubramaniam et al., 2006; Tang et al., 2007). Three large randomized trials have been conducted to evaluate the effect of inhaled NO on survival without BPD in VLBW infants (Ballard et al., 2006; Kinsella et al., 2006; Mercier et al., 2010). Only one study was able to demonstrate a modest but statistically significant benefit in survival without BPD at 36 weeks PMA (Ballard et al., 2006). Furthermore, evidence from Van Meurs et al. (2005) indicate a higher rate of mortality and intraventricular hemorrhage (IVH) in infants weighing <1000 g at birth who received inhaled NO. Large meta-analyses have since been unable to find consistent long-term improvement in mortality or the incidence and severity of BPD when using inhaled NO in preterm infants as a prevention or rescue therapy (Askie et al., 2011; Donahue et al., 2011). Currently, there remains insufficient evidence to recommend the use of inhaled NO therapy in preterm infants who have respiratory failure for the purpose of preventing or improving BPD, even in infants who have developed pulmonary hypertension (Kumar and Committee on Fetus and Newborn, 2014).

### SYSTEMIC

Sildenafil is a selective phosphodiesterase inhibitor that increases concentrations of cyclic guanosine monophosphate (GMP) and thus promotes pulmonary vasodilation. Animal studies of sildenafil have shown that it promotes alveolar growth, mitigates lung inflammation, and reduces pulmonary hypertension in hyperoxia-induced lung injury models (Ladha et al., 2005; De Visser et al., 2009). Small pilot studies have shown that sildenafil reduces pulmonary vascular pressures in infants with severe BPD with no additional side effects (Baquero et al., 2006; Mourani et al., 2009). Concerns do remain in recommending widespread use in high risk preterm neonates as an increase in mortality was found in studies of older children receiving higher doses of sildenafil (Wardle and Tulloh, 2013). However, it remains a promising therapy and further studies are needed to elucidate appropriate dose, formulation, and timing of administration in neonates with BPD (especially those with secondary pulmonary hypertension).

## LATE SURFACTANT

Historically, surfactant administration has been administered shortly after birth for the prevention and treatment of respiratory distress syndrome (RDS). While early surfactant administration has not been shown to significantly impact the development of BPD, alterations in surfactant function have been reported in older patients with a variety of chronic lung disorders, suggesting a possible benefit to late surfactant administration in the treatment of BPD (Gunther et al., 2002; Bahadue and Soll, 2012). Analysis of surfactant samples of chronically ventilated neonates suggests that this may be due to a deficiency of surfactant proteins (SP) B and C (Merrill et al., 2004). Multiple pilot trials have demonstrated an increase in tracheal SP-B concentrations and a transient improvement in oxygenation with no short-term side effects following late administration of exogenous surfactant (Merrill et al., 2011; Keller et al., 2012). A large, multicenter, blinded, RCT is currently underway in an extremely low gestational age (ELGAN) cohort examining the effects of late surfactant therapy on surfactant function and survival without BPD ^2^.

## PREVENTION STRATEGIES

### ANTIOXIDANTS

Oxygen has a unique molecular structure that is capable of accepting free electrons generated by oxidative metabolism into its outer ring. Hyperoxia, reperfusion, infection, ventilator-associated inflammation, and inadequate antioxidant defenses can produce reactive oxygen species (ROS) which are toxic to living tissues. Clinical studies suggest that ROS are involved in the pathogenesis of BPD. Plasma concentrations of ROS (allantoin, expired pentane, protein carbonyls, and 3-nitro tyrosine molecules) have been shown to be significantly elevated in the first week of life in infants developing BPD compared to infants who recover without the development of significant chronic lung disease (Ballard et al., 2008; Poggi and Dani, 2014). A strategy for antioxidant enzyme replacement was investigated by Davis et al. (1997)in high risk VLBW infants. Intratracheal administration of recombinant human CuZn superoxide dismutase (rhSOD) was associated with increased SOD levels (lung, serum, urine) and lower levels of biomarkers of acute lung injury (Rosenfield et al., 1996; Davis et al., 1997). Limited follow up data in this initial cohort did not demonstrate any difference in death, BPD, days of mechanical ventilation, oxygen requirement, or neurodevelopmental outcome (Davis et al., 2000). However, a larger trial in 302 VLBW infants followed out to 1 year corrected gestational age demonstrated a significant reduction in pulmonary morbidity (e.g., respiratory illness, emergency room visits, hospital readmissions) in the rhSOD-treatment versus the placebo group, suggesting that a reduction in early oxidant injury may still impact longer-term pulmonary outcomes (Davis et al., 2003).

### CLUB (CLARA) CELL PROTEIN (CC10)

CC10 is a 10-kilodalton protein secreted by non-ciliated bronchiolar epithelial cells (club cells) and is one of the most abundant proteins within the fluid lining the lung epithelium (Greenough, 2008). CC10 has extensive anti-inflammatory properties and has been shown to be significantly lower in tracheal aspirates of premature infants who subsequently died or developed BPD (Jorens et al., 1995; Broeckaert et al., 2000; Schrama et al., 2008). Animal studies have demonstrated that administration of recombinant human CC10 (rhCC10) upregulates SP and vascular endothelial growth factor (VEGF) expression while improving respiratory mechanics (Miller et al., 2007; Wolfson et al., 2008). A pilot trial conducted in 22 VLBW infants by Levine et al. (2005) demonstrated that intratracheal administration of rhCC10 was well-tolerated and had significant anti-inflammatory effects in the lung. No infant followed out to 6 months corrected gestational age had any significant respiratory illness following treatment with rhCC10 compared to 50% in the control group. This promising treatment is being further investigated in a multi-center randomized, blinded trial evaluating survival without long-term pulmonary morbidity (chronic respiratory morbidity at 1 year corrected age) as the primary outcome ^3^.

## CONCLUSION

Decades after initially being described by Northway et al. (1967), BPD still remains a very important complication of neonatal intensive care. BPD is a complicated multisystem disease that carries a significant physical, social, and economic burden for the survivors and their families. While multiple therapies are used routinely either alone or in combination (potentially increasing drug–drug interactions and associated side effects), there is insufficient evidence supporting short and longer-term use of many of these agents. In fact, no single therapy has been shown to have a significant impact on the incidence or severity of BPD. Targeting single mechanisms is unlikely to significantly influence BPD since it is multifactorial in nature. Future research should be focused on establishing better biomarkers predictive of BPD and associated longer-term chronic respiratory morbidity, developing stratification models to identify high-risk infants early on, and applying a multimodal approach when studying various pharmacologic interventions.

## Conflict of Interest Statement

The authors declare that the research was conducted in the absence of any commercial or financial relationships that could be construed as a potential conflict of interest.

## Footnotes

1 ClinicalTrials.gov PREMILOC trial to prevent bronchopulmonary dysplasia in very preterm neonates. Clinical.Trials.Gov identifier: NCT00623740.

2 ClinicalTrials.gov TOLSURF trial of late surfactant for prevention of bronchopulmonary dysplasia. Clinical.Trials.gov Identifier: NCT01022580.

3 ClinicalTrials.gov Efficacy of recombinant human Clara cell 10 protein (rhCC10) administered to premature neonates with respiratory distress syndrome. Clinical.Trials.gov Identifier: NCT01941745.
